# Variables Leading to Usage of Alteplase Versus Heparin Lock in Patients With Tunneled Catheters in Hemodialysis Care Project Centers, Saudi Arabia

**DOI:** 10.7759/cureus.60817

**Published:** 2024-05-21

**Authors:** Jawad Alhammouri, Debra Ockhuis, Emily Hibionada, Maram Albalawi, Roqiah Alnahdi, Basma Hikal, Elbusheer Koko, Ma Mona Lizza Alano, Manuel Troncoso, Muhammad Nauman Hashmi

**Affiliations:** 1 Hemodialysis Care Project, Ministry of National Guard Health Affairs, Riyadh, SAU; 2 Research Office, King Abdullah International Medical Research Center, Riyadh, SAU; 3 Research Office, King Saud Bin Abdulaziz University for Health Sciences, Riyadh, SAU; 4 Biostatistics, King Abdullah International Medical Research Center, Riyadh, SAU; 5 Hemodialysis Care Project, Ministry of National Guard Health Affairs, Jeddah, SAU; 6 Hemodialysis Care Project, Ministry of National Guard Health Affairs, Madinah, SAU; 7 Hemodialysis Care Project, Ministry of National Guard Health Affairs, Makkah, SAU; 8 Hemodialysis Care Project, Ministry of National Guard Health Affairs, Hail, SAU; 9 Nephrology, Ministry of National Guard Health Affairs, Jeddah, SAU

**Keywords:** recombinant tissue plasminogen activator (rt-pa), heparin, variables, thrombosis, catheter dysfunction, catheter locking solution, alteplase, hemodialysis, central venous catheter (cvc), tunneled catheter

## Abstract

Introduction: A hemodialysis tunneled catheter is one option for vascular access used with hemodialysis patients; however, catheter complications such as thrombosis are still inevitable. To prevent thrombosis formation, a catheter-locking solution is instilled between dialysis sessions. Heparin is used as a default locking solution in our Hemodialysis Care Project centers, while a recombinant tissue plasminogen activator (rt-PA) such as alteplase is used to treat suspected catheter thrombosis. This study aimed to identify the clinical factors, catheter brands, and hemodialysis variables that influence the choice of use for alteplase versus heparin, for those patients with tunneled catheters, and reduce overprescribing of high-alert medication alteplase.

Methods: A retrospective medical chart review study was conducted involving 230 patients with tunneled catheters; the first group of 133 patients used alteplase regularly three times a week, while the second group of 97 patients completed at least one year using the same catheter access with heparin lock only.

Results: Multivariate logistic regression and logistic regression analysis showed a significant association (p < 0.05) between different variables. Results suggest that overweight and hyperlipidemia patients are more likely to use alteplase. Patients using brand-name catheters such as Hemostar/Vas-cath (BD, Franklin Lakes, NJ) are less likely to use heparin than those using Medcomp catheters (Medcomp, Yuma, AZ). In addition, patients having a history of angioplasty would be less likely to have heparin than no angioplasty. Moreover, if the patient’s fluid removal were equal to or less than 2 kg, they would be more likely to use heparin and vice versa.

Conclusion: The study postulates that identified variables affect whether alteplase or heparin is used in hemodialysis tunneled catheters, and may be useful to increase awareness, improve practices, or judiciously control the use of alteplase within Saudi Arabia and globally.

## Introduction

Patients with hemodialysis (HD) tunneled catheters (used interchangeably with the term central venous catheters [CVCs]) require patency and good blood flow to receive optimal HD treatment [1]. The use of CVCs is common in HD, and complications such as CVC dysfunction can arise, leading to reduced dialysis adequacy. According to the 2020 Kidney Disease Outcomes Quality Initiative (KDOQI) guideline, CVC dysfunction is defined as the failure to maintain the prescribed extracorporeal blood flow required for adequate HD without lengthening the prescribed HD treatment. The KDOQI Vascular Access Guideline Work Group considers a sufficient extracorporeal blood flow to be 300 ml/min [2]. Approximately 50% of HD catheters fail within one year [3]; up to two-thirds of the failures are due to thrombosis [4-5].

Practically, the initial method for the treatment of a dysfunctional CVC is conservative, i.e., checking for mechanical obstruction (e.g. kinks under catheter clamps at the exit site, positioning of the patient) and forcefully flushing the lumens with normal saline 0.9%. If conservative measures fail, and a thrombus is suspected, the administration of a thrombolytic agent is needed to restore the function of a dysfunctional CVC. Alteplase, a recombinant tissue plasminogen activator (rt-PA), is the thrombolytic catheter-locking agent used to treat CVC dysfunction in our Hemodialysis Care Project centers.

The CVC lumens are instilled with a catheter-locking solution after each HD session until the next session to prevent thrombosis. Heparin has been the traditional locking solution; evidence supporting the use of various locking solutions to achieve these objectives is limited. Several small studies have assessed whether citrate and heparin are equally efficacious for maintaining catheter patency, [6-7] but the interpretation of the results is limited because the studies had a short follow-up period and included both uncuffed and cuffed CVC [8].

The optimal management of an occluded dialysis catheter remains unclear. Thrombolytic agents are commonly used as first-line treatment for occluded HD catheters [9]. Chronic use of alteplase is recommended in specific circumstances, namely, substituting one weekly dose with alteplase or if CVC is the patient’s last option for HD viable access [10].

However, alteplase is an expensive medication (2 mg = 68$; 4 mg = 130$)[11], in addition, it is associated with a spectrum of side effects, the most serious and common of which is bleeding, therefore its use and dosage must be limited to the absolute necessities.

## Materials and methods

This study employed a non-probability purposive sampling method and used retrospective chart review for data collection. The sample size was determined by considering all adult patients who had only tunneled catheters (intrajugular or femoral) and underwent regular HD three times a week before September 30, 2022. The study was conducted at six adult Hemodialysis Care Project centers (Riyadh (North and South), Hail, Jeddah, Makkah, and Medina) of the Ministry of National Guard Health Affairs in the Kingdom of Saudi Arabia.

This study explored two main groups of patients with specific inclusion and exclusion criteria. The first group consisted of patients on regular HD three times a week using alteplase regularly for at least one month in every HD session before the selected date of September 30, 2022. Excluded from this group were patients who had a catheter exchange, or discontinued alteplase for any reason. The second group of patients were those on regular HD three times a week who used the same tunneled catheter access with only heparin lock regularly for at least one year in every HD session before the selected date of September 30, 2022. Excluded from this group were patients who had a history of alteplase lock or dwell in the last 12 months, or if they had catheter exchange or removal for any reason.

**Figure 1:**
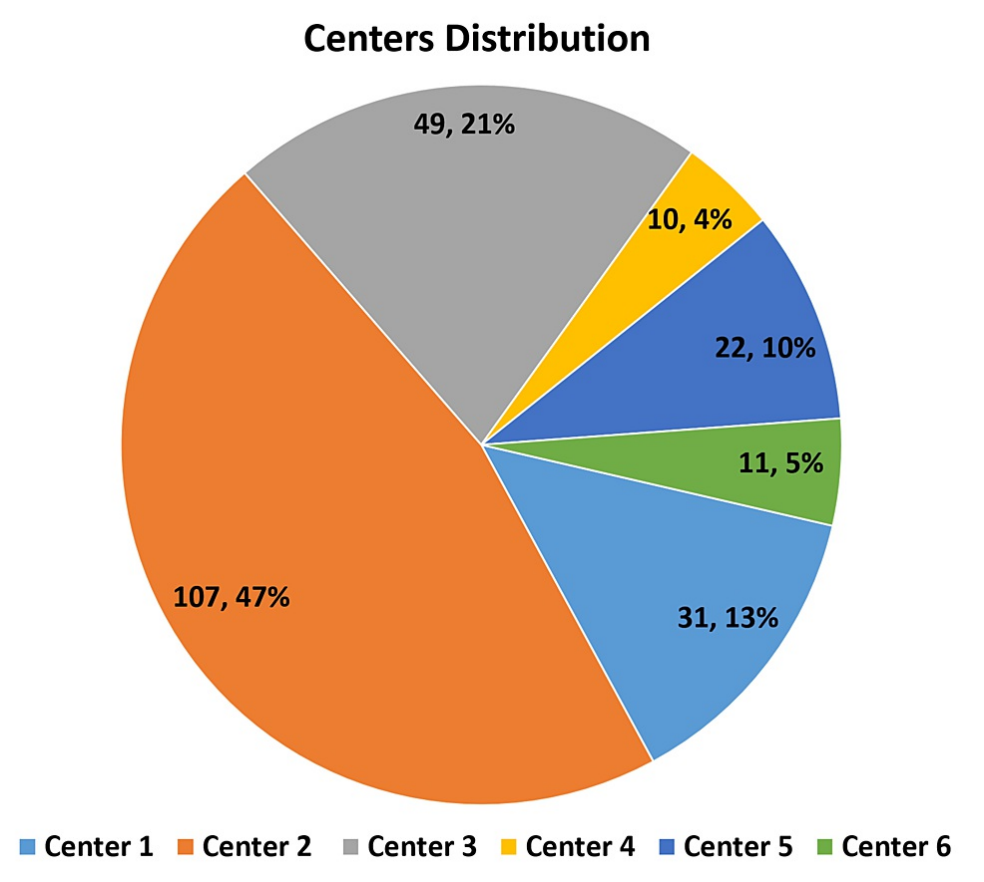
Distribution of the population under study

Out of 600 patients with tunneled catheters, 230 patients met the study’s inclusion criteria (Figure 1).

Instrumentation

The data collection form (Appendix 1) was developed based on the study’s aims and objectives and approved by the research team. Pilot testing of the self-administered data collection form was conducted to ensure that any problems concerning the completion of the form were identified and addressed before starting the study. The data were extracted from the electronic medical record by sub-investigators, overseen by study coordinators and the principal investigator.

The coding of raw data such as human age classification, body weight, and hypertension done according to the World Health Organization criterion and thereafter sent to the King Abdullah International Medical Research Center (KAIMRC) statistician for analysis.

SAS software version 9.4 (SAS Institute Inc., Cary, NC) was used to statistically analyze the collected data. In addition, the chi-squared test was used to assess the association between categorical variables when appropriate. Logistic regression and multivariate logistic regression analysis were performed for some variables.

This study adhered to the Principles of the Helsinki Declaration (World Medical Association 2008). The Ministry of National Guard Health Affairs Nursing Research Committee and the Institutional Research Board (IRB) of King Abdullah International Medical Research Center approved the study (IRB/2538/22).

A potential benefit of this study is providing data from specific Saudi Arabia HD centers perspectives and this data will contribute to and extend existing knowledge in determining whether contributing factors are instrumental in using alteplase versus heparin lock or if alteplase is overprescribed unnecessarily without in-depth root cause analysis within Saudi Arabia and globally.

## Results

In this study, males comprised 114 (49.6%) and females, 116 (50.4%). Using WHO age classification, the distribution of the population consisted of the following: a younger people age group of 18-65 years (n=151; 65.65%); a middle-aged group of 66-79 years (n=59; 25.65%) and an elderly/senior group of 80+ years (n=20; 8.70%). Alteplase medication was prescribed to 133 (57.83%) patients and heparin to 97 (42.17%).

**Figure 2:**
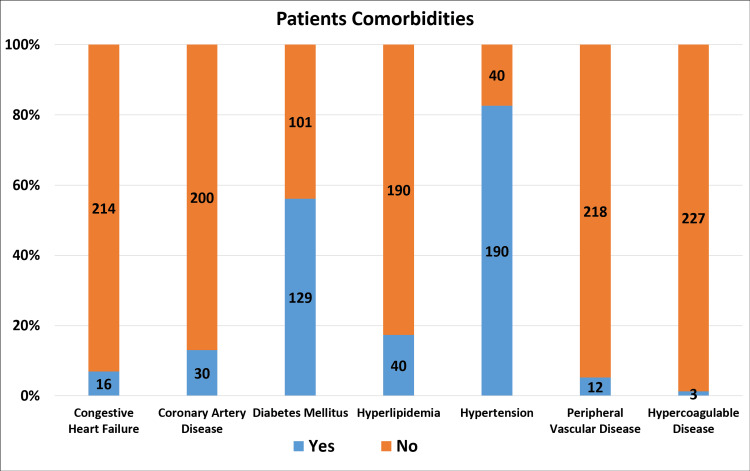
Patients comorbidities result by number of patients (n=230)

This study employed descriptive statistics to determine the frequency, percentage, and distribution of different variables and a p-value less than 0.05 indicated a statistically significant result (Table 1). Presented in Figure 2 are descriptive data such as patient comorbidities.

The various catheter brands included in the study include: Angiodynamics (AngioDynamics Inc., Latham, NY), Coviden/Mahurkar (Meditronic PLC, Dublin, Ireland); Glidepath (Becton, Dickinson and Company (BD), Franklin Lakes, NJ); Hemostar/vas-cath (BD); Medcomp (Medcomp Inc., Yuma, AZ).

**Table 1:** Descriptive statistics variables of the association group (alteplase vs. heparin) Total: n=230; alteplase: n=133; heparin: n=97 *Chi-squared test

Variable	Frequency (n)	Percent ( %)	Alteplase; n(%)	Heparin; n(%)	P-value
BMI					0.0117*
Underweight (<18.5)	18	7.83	6 (4.51)	12 (12.37)	
Normal range (18.5-24.9)	62	26.96	29 (21.80)	33 (34.02)	
Overweight (25-29.9)	76	33.04	50 (37.59)	26 (26.80)	
Obese (>=30)	74	32.17	48 (36.09)	26 (26.80)	
Catheter brand name					0.0017*
Angiodynamics	50	21.74	28 (21.05)	22 (22.68)	
Coviden/Mahurkar	10	4.35	5 (3.76)	5 (5.15)	
Glidepath	82	35.65	38 (28.57)	44 (45.36)	
Hemostar/vas-cath	50	21.74	40 (30.08)	10 (10.31)	
Medcomp	14	6.09	5 (3.76)	9 (9.28)	
Others	24	10.43	17 (12.78)	7 (7.22)	
Catheter lumen priming volume in ml (A: arterial V: venous)					0.0120*
A1.6 V1.6	86	37.39	40 (30.08)	46 (47.42)	
A1.6 V1.7	48	20.87	31 (23.31)	17 (17.53)	
A1.8 V1.9	17	7.39	15 (11.28)	2 (2.06)	
A1.9 V2.1	43	18.7	22 (16.54)	21 (21.65)	
A2.1 V2.2	10	4.35	7 (5.26)	3 (3.09)	
Others	26	11.3	18 (13.53)	8 (8.25)	
History of angioplasty					0.0022*
No	203	88.26	110 (82.71)	93 (95.88)	
Yes	27	11.74	23 (17.29)	4 (4.12)	
Hyperlipidemia					0.0387*
Yes	40	13.39	29 (21.80)	11 (11.34)	
No	190	86.61	104 (78.20)	86 (88.66)	
Average of fluid removal (kg)					0.0057*
=<2 kg	113	49.13	55 (41.35)	58 (59.79)	
>2 kg	117	50.87	78 (58.65)	39 (40.21)	

In the study result, if a patient is more likely to be alteplase-dependent due to a certain variable, they are less likely to be heparin-dependent and vice versa.

Thereafter, multivariate logistic regression analysis was done to determine the association between dependent and independent variables. Multivariate logistic regression analysis (Table 2) showed a significant association between BMI, hyperlipidemia, and the heparin vs. alteplase group (p < 0.05). In the BMI variable, we found that participants who were in the normal range and underweight were more likely to have heparin than overweight participants. With the hyperlipidemia variable, we found that participants with hyperlipidemia were less likely to have heparin.

**Table 2:** Multivariate logistic regression analysis examining the relationship between BMI, hyperlipidemia, and Heparin vs. Alteplase group *(p < 0.05)

Dependent variable	Independent variable	Adjusted odd ratio (AOR)	95%CI for AOR		P-value
			Lower	Upper	
Heparin vs. Alteplase group	BMI normal range vs. overweight	2.265	1.119	4.585	0.0231*
	BMI obese vs. overweight	1.119	0.561	2.231	0.7491
	BMI underweight vs. overweight	4.129	1.351	12.617	0.0128*
	Hyperlipidemia (yes vs. no)	0.45	0.206	0.985	0.0457*

Table 3 showed a significant association between the average fluid removal in kg and the Heparin vs. Alteplase group. In the average of fluid variables, our references were greater than 2 kg, and we found that fluid removal equal to or less than 2 kg was more likely to have heparin.

**Table 3:** Logistic regression analysis examining the relationship between the average of fluid removal (kg) and the Heparin vs. Alteplase group *(p < 0.05)

Dependent variable	Independent variable	Odd Ratio (OR)	95%CI for OR		P-value
			Lower	Upper	
Heparin vs. Alteplase group	Average of fluid loss (=<2 kg as per NKF vs. >2 kg)	2.109	1.238	3.593	0.0060*

Regarding the catheter brand-name variable (Table 4), our reference was the Glidepath brand, and we found that patients using Hemostar/Vas-cath were less likely to have heparin than those using the Glidepath catheters, but patients using Medcomp were more likely to have heparin than those using Glidepath. However, there is no significant association between other brands and Glidepath. In addition, for the angioplasty history variable, our reference was “No” angioplasty, and we found that if there was a history of angioplasty, these patients were less likely to be on heparin than those without a history of angioplasty.

**Table 4:** Multivariate logistic regression analysis examining the relationship between catheter brand name, history of angioplasty, and the Heparin vs. Alteplase group *(p < 0.05)

Dependent variable	Independent variable	Adjusted odd ratio (AOR)	95%CI for AOR		P-Value
			Lower	Upper	
Heparin vs. Alteplase group	Angiodynamics vs. Glidepath	0.810	0.236	2.782	0.7379
	Coviden/Mahurkar vs. Glidepath	2.488	0.507	12.207	0.2613
	Hemostar/vas-cath vs. Glidepath	0.379	0.149	0.960	0.0408*
	Medcomp vs. Glidepath	4.451	1.096	18.080	0.0368*
	Others vs. Glidepath	0.705	0.206	2.410	0.5778
	No of angioplasty Yes vs. No	0.183	0.055	0.609	0.0056*

Lastly, with the arterial and venous priming volume variable Table 5, our reference volumes (in ml) were A1.6 V1.6, and we found that A1.6 V1.7 and A1.8 V1.9 were less likely to have heparin than A1.6 V1.6. However, there was no significant association between A1.9 V2.1, A2.1 V2.2, and A1.6 V1.6.

**Table 5:** Logistic regression analysis of the relationship between arterial and venous priming volume, and the Heparin vs. Alteplase group *(p < 0.05)

Dependent variable	Independent variable catheter lumen priming volume (ml)	Odd ratio (OR)	95%CI for AOR		P-value
			Lower	Upper	
Heparin vs. Alteplase Group	A1.6 V1.7 vs. A1.6 V1.6	0.477	0.230	0.987	0.0461*
	A1.8 V1.9 vs. A1.6 V1.6	0.116	0.025	0.538	0.0059*
	A1.9 V2.1 vs. A1.6 V1.6	0.830	0.399	1.727	0.6183
	A2.1 V2.2 vs. A1.6 V1.6	0.373	0.090	1.538	0.1723
	Others vs. A1.6 V1.6	0.386	0.152	0.984	0.0461*

## Discussion

Despite several studies showing that using an rt-PA as a locking solution for HD catheters significantly decreases catheter-related bacteremia and catheter dysfunction, enhances catheter blood flow, and prevents clot formation as compared with heparin, heparin remains the standard of care due to its efficacy, reasonable side effect profile, and cost-effectiveness. The evidence to guide the selection of the optimal catheter-locking solution in the primary prevention of catheter malfunction is limited [12]. Catheter thrombosis occurs at a frequency of 0.5 to 3.0 events per 1000 catheter days, resulting in shortened dialysis treatments, less-than-adequate dialysis, and increased morbidity and mortality.

Our study analysis suggests that patients’ physical characteristics like BMI and hyperlipidemia increased the risk of using alteplase. In 2018, Kosa et al. [13] found that a shorter time to first rt-PA administration in all patient’s catheters was associated with increased body mass index. Our search of PubMed articles found no studies over the last 10 years that investigated the impact of hyperlipidemia on thrombus formation or the use of rt-PAs for HD catheters. The principal investigator questions whether alteplase is the correct medicine to treat and improve catheter dysfunction when the patient has risk factors (overweight and hyperlipidemia) that could affect the patient's vessels like atherosclerosis.

Most HD patients need to limit their fluid intake to approximately 32 ounces per day. The goal for an average-sized patient is to keep fluid weight loss at or below 1 kg (2.2 pounds) each day, this equals 2 kg (4.4 pounds) fluid weight loss when there are two days between treatments and 3 kg (6.6 pounds) when there are three days between treatments [14]. The patient who loses more than 2 kg every treatment through ultrafiltration would be at risk for alteplase dependence. This phenomenon regarding the effects and/or mechanism of fluid removal on patients leading to the use of alteplase should be studied further as no research was found on this subject. 

Providing sufficient blood flow through the dual-lumen CVC for HD depends on the size of the catheter, position, and direction of the catheter tips, design of the tips, and number and size of the side holes around the tip [15-16]. Despite catheter materials being biocompatible and hemocompatible, complications due to thrombosis and infection are still inevitable. The catheter brand seems to play a significant role in leading to the use of alteplase to give a reliable performance rather than the use of heparin. In 2014, Van Der Meersch et al. [17] in a randomized controlled trial found that the Palindrome catheter (Medtronic PLC) was less thrombolytic and achieved higher blood flow rates than the HemoStar catheter brand, therefore, key personnel involved in purchasing HD catheters should ensure all aspects of the catheters' durability are evaluated before purchase and surveillance or evaluation for each catheter brand from time to time is highly recommended.

KDOQI suggests that the clinician's discretion and best clinical judgment be considered regarding the choice of tunneled HD CVC type and design [2]. The study result showed significant statistical results about catheter lumen priming volume and we found that the catheter priming volumes A1.6 V1.7 and A1.8 V1.9 are correlated co-incidentally to the same catheter brand HemoStar, which is less likely to have heparin. The investigator inquires whether alteplase is the correct solution for treatment with respect to the quality of catheters utilized by different referral hospitals.

Treatment options for venous stenosis are limited [18], however, those patients with recurrent angioplasty procedures would be more likely to use alteplase than those who did not undergo angioplasty. Previous studies have suggested a high incidence of restenosis after coronary angioplasty performed in patients with renal failure [19]. Hence, angioplasty procedures should be limited if they are not clinically indicated as this may increase the probability of requiring alteplase.

The perspectives of the investigator and sub-investigators are: the cost of the catheter is not only related to the price of each unit but other effects must be considered such as patient outcome, unplanned hospitalization, medications, and staffing.

More clinical studies are needed to identify the ideal HD CVC that meets the criteria of long survival times, low CVC complication rates (dysfunction and CRBSI), and potentially lower patient complication rates (vascular stenosis and thrombosis). We hope this study's results will contribute to and extend existing knowledge in determining whether contributing factors are instrumental in using alteplase versus heparin lock or if alteplase is overprescribed unnecessarily without in-depth root cause analysis.

Limitations

This study was performed retrospectively, so limitations encountered were lack of adequate documentation for some patients’ records and limited access to different primary hospitals to review patients’ medical records. Also, there were missing laboratory blood results for some patients due to assorted reasons and, in some patients’ records, evidence of areas lacking consistent documentation standards. Lastly, an absence of clear systemic or standard updates for patients’ records and history (comorbidities, home medications).

Recommendations

This project aimed to reduce the use of alteplase for patients with HD CVCs in Hemodialysis Care Project centers in Saudi Arabia. To achieve this, we recommend discussing the research outcomes and risk factors with HD healthcare workers and decision-makers. Alteplase or heparin lock for HD patients with CVCs should be individualized and monitored by the nephrologist and the dialysis team and adjusted according to the patient’s clinical condition and response. Also, using the best brand of HD CVC that has long-term functioning with the lowest rate of complications has a profound positive impact on individual patient care and the cost of dialysis catheter management. Continuous tracking of the patients’ catheter performance is highly recommended when receiving a patient from different primary hospitals. Developing a clear protocol or procedure for administering alteplase to prevent HD tunneled catheter malfunction and designing a tool for monitoring and tracking alteplase use is also recommended. Additionally, a standard database program or folder could be set up to store and retrieve the patient’s records and history to facilitate data collection and reporting. Lastly, we recommend that a randomized clinical trial be conducted to support or change the current results and knowledge of the effects of rt-PAs and heparin for locking HD tunneled catheters.

## Conclusions

Some HD patients with CVCs have been using heparin lock for years without ever needing alteplase, as in the case of the 97 patients visiting Hemodialysis Care Project centers in our study. On the other hand, 133 patients still use alteplase as a dwell and a lock for their catheter even though they have had several catheter exchanges. We assume that some of the variables that we studied increase the risk of some HD patients becoming dependent on alteplase to improve catheter performance.

Through this study, we aimed to identify, and expand the existing knowledge on the collected variables that could influence the use of alteplase or heparin as catheter locks. Healthcare workers in Hemodialysis Care Projects (physicians, nurse managers, nurses, and pharmacists) need to look at these variables when dealing with malfunctioning tunneled catheters as some of these variables are preventable (catheter brand) and others can be managed (BMI, hyperlipidemia, weight gain).

## Figures and Tables

**Figure 3:**
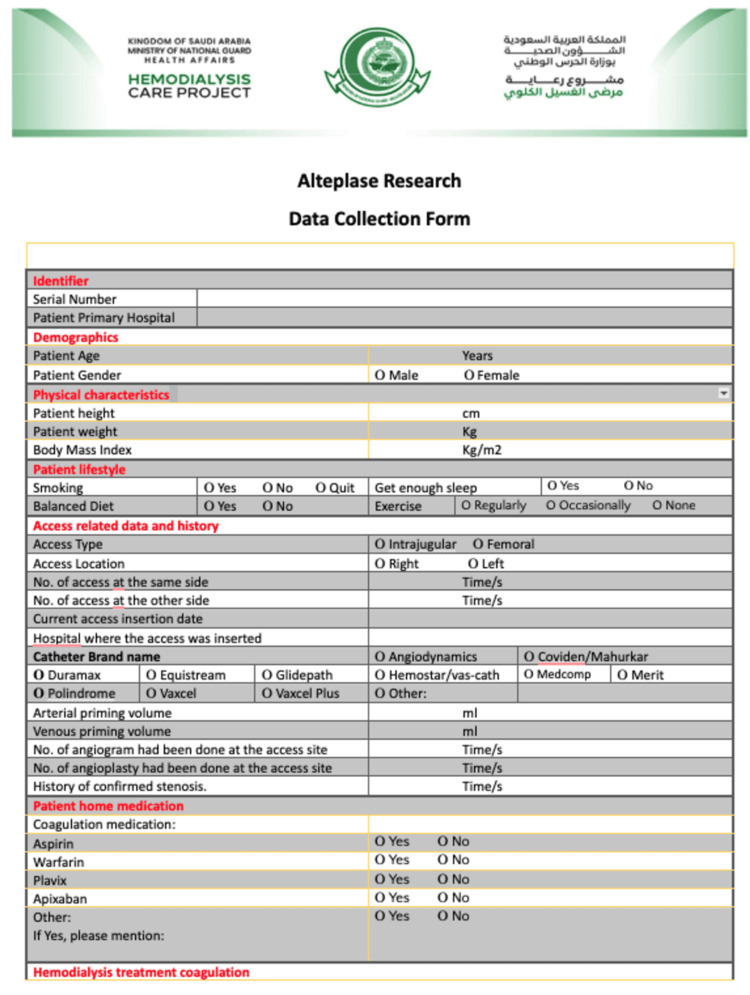
Data Collection Page 1

**Figure 4:**
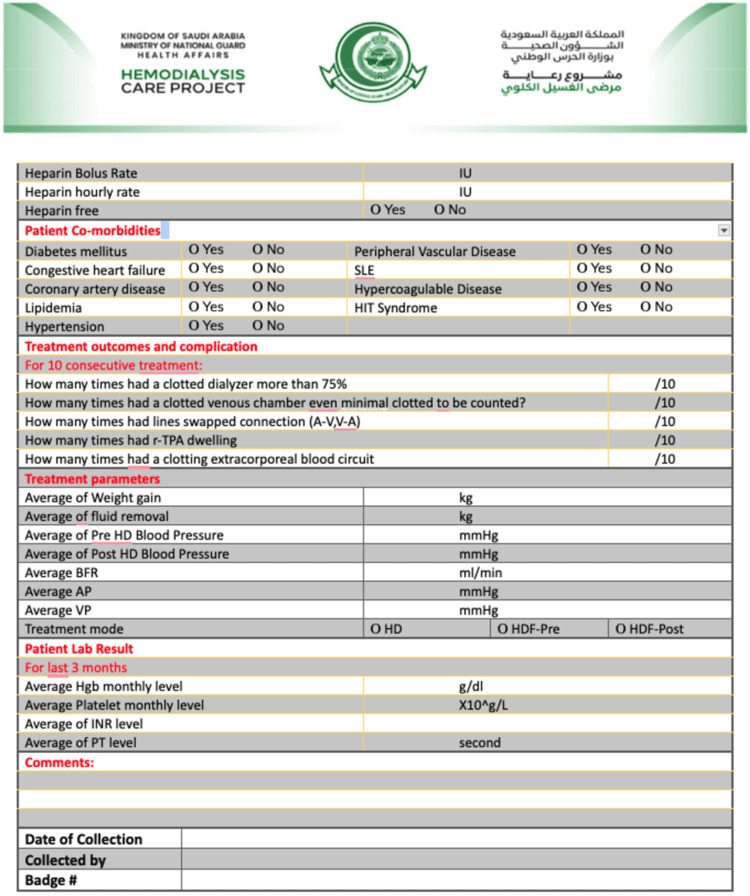
Data Collection Page 2

## Appendices

Appendix 1

Figures 3-4 show the forms used for data collection.
